# Converting spring-born heifers into summer-calving herds increases subsequent cow longevity and productivity

**DOI:** 10.1093/tas/txaf077

**Published:** 2025-06-11

**Authors:** Tim Goodnight, Jacki A Musgrave, Kacie L McCarthy, J Travis Mulliniks

**Affiliations:** Department of Animal Science, University of Nebraska-Lincoln, Lincoln, NE 68583, USA; West Central Research and Extension Center, University of Nebraska, North Platte, NE 69101, USA; Department of Animal Science, University of Nebraska-Lincoln, Lincoln, NE 68583, USA; Department of Animal and Rangeland Sciences, Oregon State University, Corvallis, OR 97331, USA

**Keywords:** heifer development, longevity, long-term productivity, reproductive performance

## Abstract

The objective of this study was to determine the impact of converting spring-born heifers into a summer-calving herd on growth, reproductive performance, longevity, and productivity compared to early or late-born May heifers. Over a 3-yr period, 273 Red Angus/Simmental crossbred heifers were utilized to determine the impact of converting March-born heifers (**Convert**; n = 90) to a May-calving herd compared to May-born heifers on reproductive performance, body weight, and calf performance from a yearling to 5 yr of age. May-born heifers were retrospectively grouped into 2 different groups: heifers born in the first 21-d of calving as a heifer calf (**Early**; n = 123) or heifers born after the 21-d of calving as a heifer calf (**Late**; n = 60). Heifers were exposed to bulls for a 45-d breeding season with a bull-to-heifer ratio of 1:20. Heifers were synchronized with a single injection of prostaglandin F2α (5-mL i.m.; Lutalyse, Zoetis, Parsippany, NJ) 5-d after bulls were introduced in the pasture for breeding. Data were analyzed as a randomized design using the MIXED procedure (SAS Inst. Inc., Cary, NC, USA). All binomial data were analyzed using PROC GLIMMIX. Heifer served as the experimental unit with treatment and year developed set as fixed effects. Heifer body weight (**BW**) at December, May, July, and pregnancy check (Oct) were greater (*P* < 0.01) for Convert heifers with no difference between Early and Late born heifers. Heifer body condition score (**BCS**) at pregnancy check was not different (*P* = 0.13) among heifer treatment groups. However, pregnancy rate tended (*P* = 0.08) to increase for Convert heifers with no difference between Early and Late born May heifers. After 5 yr of age, retention rate was increased (*P* = 0.05) in Convert cows compared to Early and Late May-born cows with no difference (*P* = 0.64) between the two May-born heifer groups. Total kilograms of calf weaned was greater (*P* = 0.04) in Convert cows than their counterparts with no differences (*P* = 0.94) between Early and Late May-born cows. This study implies that selecting replacement heifers from an earlier calving season for replacements in a later calving season increases the longevity and long-term productivity of the cowherd than selecting heifers within the same calving season. This may be even more important in resource-poor breeding environments that create reproductive challenges for heifers and rebreeding young cows.

## Teaser

Strategies to increase longevity and productivity of the cow herd is warranted. In this study, selecting older heifers from an earlier calving herd increases cow herd retention and overall productivity up to 5 yr of age compared to retaining heifers within the cow herd in semi-arid environments.

## INTRODUCTION

Developing replacement heifers is one of the most expensive and complex management decisions for a cow-calf producer, which also has long-term implications for profitability (McFarlane et al., 2018). Traditionally, cow-calf producers have been recommended to develop weaned heifers to reach a certain percentage of mature BW (i.e., 65%) before the start of the breeding season to maximize pregnancy rate (Patterson et al., 1992). Developing heifers at this target BW can be a significant cost to cow-calf producers, which is primarily associated with nutritional costs (i.e., grazing, harvested feed, supplements). However, reducing heifer investment costs while maintaining reproductive performance is important for profitability due to the number of calf crops required to pay for development costs, which can be up to 10 yr of age (McFarlane et al., 2018).

Heifer selection and nutritional strategy during the development phase can have a major impact on future productivity and longevity in the cowherd. For instance, increasing metabolizable protein supply by increasing the proportion of rumen undegradable protein in supplements fed to yearling heifers on dormant forage before breeding increased pregnancy rates, longevity, and net return compared with heifers developed in a dry lot (Mulliniks et al., 2013). In addition, heifers that calve earlier in the calving season have been shown to have increased lifetime productivity (Cushman et al., 2013).

Season of calving and the subsequent environmental conditions during the breeding season do impact heifer reproductive performance. For instance, Springman et al. (2018) reported greater pregnancy rates in March-born heifers compared to May-born heifers in their respective breeding seasons (May vs July). Selecting replacement heifers from earlier calving seasons may provide the ability to increase reproductive performance and longevity; however, older heifers from less harsh breeding seasons may not be adapted and have better fitness for the more resource-poor breeding season. Therefore, the objective of this study was to determine the impact of converting spring-born heifers into a summer-calving herd on growth, reproductive performance, longevity, and productivity.

## MATERIALS AND METHODS

The Institutional Animal Care and Use Committee at the University of Nebraska-Lincoln (IACUC approval number 1474) approved animal procedures and facilities used in this experiment.

In a 3-yr study, 273 Red Angus/Simmental crossbred heifers were utilized to determine the impact of converting March-born heifers (**Convert**; n = 90) into a May-calving herd compared to May-born heifers on reproductive performance, body weight, calf performance, and long-term productivity. Heifers used in this study were born in 2017 to 2019. May-born heifers were retrospectively grouped into 2 different groups: heifers born in the first 21-d of calving as a heifer calf (**Early**; n = 123) or heifers born after the first 21-d days of calving as a heifer calf (**Late**; n = 60). The percentage of heifers calving within the first 21 d of calving was calculated after 2 or more heifers had calved. The May-calving herd was initiated in 2009 and developed from moving March-calving cows into the May-calving herd. In addition, the same bulls were utilized in both herds during breeding.

After weaning each year, Convert and both May-born heifer groups were managed together and grazed upland range pastures continuously. Heifers were fed twice weekly dried distillers grains (**DDGS**, dry matter (**DM**) basis) at a rate of 0.68 kg/heifer daily from January to April with no additional supplement given during the breeding season. The DDGS was 32% CP with an assumed total digestible nutrients (TDN) of 108% (Loy et al., 2008; DM basis). Heifer BW was collected at weaning, prebreeding, pregnancy diagnosis, and prior to calving. Heifer BCS was also collected at pregnancy diagnosis and pre-calving by two experienced technicians using visual appraisal and palpation. Percent of mature BW at breeding as a yearling heifer was estimated by the average cow BW at 5 yr of age for each treatment group. Heifers were exposed to bulls for a 45-d breeding season with a bull-to-heifer ratio of 1:20 starting in mid-July each year. Heifers were synchronized with a single injection of prostaglandin F2α (5-mL i.m.; Lutalyse, Zoetis, Parsippany, NJ) 5 d after bulls were introduced in the pasture for breeding. Pregnancy diagnosis was conducted approximately 40 d after the end of breeding season via transrectal ultrasonography (ReproScan, Beaverton, OR).

Two- and 3-yr-old cows were managed as one group and mature cows were managed as a group. However, overall management were similar across the different age group herds. Cows were exposed to fertile bulls in August for a 45-d breeding season. Approximately 45 d before breeding in each herd, cows received prebreeding vaccinations (Vista 5 VL5 SQ; Merck, Kenilworth, NJ). Each year, pregnancy diagnosis was determined approximately 75 to 110 d after the end of breeding season at weaning in December by transrectal ultrasonography.

At birth, calves received a seven-way clostridial vaccine (Alpha 7, Boehringer Ingelheim, Duluth, GA). At branding, calves were vaccinated for infectious bovine rhinotracheitis, bovine viral diarrhea types I and II, bovine parainfluenza virus-3, bovine respiratory syncytial virus, *Mannheimia haemolytica*, and *Pasteurella multocida* (Vista Once SQ, Merck, Kenilworth, NJ) and bull calves were castrated. In addition, a seven-way clostridial vaccine was also given at branding (Vision 7, Merck, Kenilworth, NJ). At weaning, calves received one vaccination of Vista Once SQ with a second dose occurring 14 d later. A seven-way clostridial vaccine with somnus (Vision 7 Somnus, Merck, Kenilworth, NJ) was also given at weaning. Calf BW was measured at birth, prebreeding, and weaning each year. An adjusted 205-d BW was calculated without adjusting for cow age or sex of calf.

Percent of the originally developed replacement heifers remaining in the herd after pregnancy check each year was recorded to determine the yearly retention rate. Cows were removed from the herd if they failed to wean a calf or were determined to be non-pregnant.

Native range forage samples were collected in June, July, September, and December each year with esophageal fistulated beef cows. Before collection, 3 esophageal cows were held off feed and water for 12 hour. At the time of collection, esophageal plug was removed, solid bottom nylon bags were placed around each cow’s neck, and cows were allowed to graze for approximately 30 min. Masticate and saliva collected in each bag were collected and lyophilized using a Vertis Freezemobile 35 XL laboratory lyophilizer (SP Scientific, Gardiner, NY). After freeze-drying, a subsample for each cow was analyzed for nutrient content using wet chemistry procedures at a commercial laboratory (Ward Laboratory, Kearney, NE). Nutrient composition of the native range across the months and years of the study is summarized in Table 1.

**Table 1.** Nutritional composition (% DM) of native range in each year of the study

Item		CP	TDN
2017	June	12.0	62.6
	July	7.2	57.2
	Sept	7.7	56.9
	Dec	5.3	55.9
2018	June	10.4	62.7
	July	7.5	60.3
	Sept	6.2	61.4
	Dec	6.0	53.0
2019	June	11.1	62.1
	July	10.7	57.3
	Sept	8.9	60.6
	Dec	7.9	60.0
2020	June	9.4	58.2
	July	7.7	65.6
	Sept	7.0	56.9
	Dec	4.4	53.6
2021	June	10.4	60.7
	July	11.9	55.5
	Sept	6.3	51.3
	Dec	5.2	49.0
2022	June	11.7	58.0
	July	10.1	55.3
	Sept	9.1	56.5
	Dec	5.4	53.4
2023	June	10.7	62.0
	July	8.7	51.4
	Sept	6.2	51.4
	Dec	6.8	55.1

### Statistical Analysis

A completely randomized experimental design was used and data were analyzed using the MIXED procedure (SAS Institute Inc., Cary, NC). The initial model included the fixed effects of treatment, year, and the interaction of treatment × year. Interactions that were not significant were removed from the model. All binomial data were analyzed using PROC GLIMMIX. The model included fixed effects of treatment, year, calf sex, and their interaction. Least squares means and SEM for binomial data were obtained using the ILINK function. Significance level was set at *P* ≤ 0.05 and as a tendency if *P* > 0.05 and *P* ≤ 0.10.

## RESULTS

### Impact on Heifer Performance

As designed and expected, heifer birth date was different (*P* < 0.01; Table 2) among the different heifer groups with Convert heifers being the oldest and Late heifers the youngest. Heifer BW at December, May, July, and pregnancy check (Oct) was greater (*P* < 0.01) for Convert heifers with no difference (*P* ≥ 0.42) between Early and Late born May heifers. Heifer BCS at pregnancy check was not different (*P* = 0.13) among heifer treatment groups. Pregnancy rate; however, tended (*P* = 0.08) to increase for Convert heifers with no difference (*P* = 0.88) between Early and Late born May heifers.

**Table 2.** Impact of converting March-born heifers in a May-calving herd on yearling heifer performance

	Treatment^1^				
Item	Convert	Early	Late	SEM	*P*-value
n =	90	123	60	--	--
Date of Birth, Julian day	73^a^	132^b^	155^c^	1	< 0.01
Heifer BW, kg					
December	227^a^	200^b^	200^b^	2	< 0.01
May	276^a^	248^b^	251^b^	4	< 0.01
July^2^	346^a^	315^b^	313^b^	4	< 0.01
Pregnancy check^3^	392^a^	372^b^	362^b^	5	< 0.01
Percentage of mature BW^4^, %	74^a^	65^b^	64^b^	1	< 0.01
Heifer BCS					
Pregnancy check^3^	5.9	5.8	5.8	0.04	0.13
Pregnancy rate, %	88^a^	80^b^	81^b^	--	0.08

^a,b,c^Means with different superscripts differ (*P* < 0.05).

^1^Treatments = March-born heifers converted over into a May-calving herd (Convert), May-born heifers born in the first 21-d of the calving season (Early), and May-born heifers born after the first 21-d of the calving season (Late).

^2^July BW = prebreeding BW.

^3^Pregnancy checking occurred in October each year.

^4^Estimated percentage of mature BW at the start of breeding (July).

### Impact on Performance as a 2-yr-Old

Calving date as a 2-yr-old was not influenced (*P* = 0.21; Table 3) by heifer age treatment groups. Convert heifers were larger (*P* < 0.01) at pre-calving and pre-breeding as a 2-yr-old with no difference (*P* ≥ 0.52) between Early and Late May-born heifers. At weaning, Convert cows tended (*P* = 0.10) to have greater BW than Early and Late May-born cows. Pre-calving BCS was greater (*P* < 0.01) for Convert cows with no difference (*P* = 0.72) between Early and Late May-born cows. However, BCS was not different (*P* ≥ 0.29) at pre-breeding and weaning among treatment groups. Calf BW at birth was not influenced (*P* = 0.65) by previous treatment groups. However, calves born from Convert cows did have greater (*P* < 0.01) pre-breeding and weaning BW than calves from Early and Late May-born cows. In addition, Early May-born cows did have a tendency (*P* = 0.10) to wean a larger calf than Late May-born cows. Adjusted 205 d calf BW was influenced (*P* < 0.01) by treatment groups. Calves from Convert cows had the greatest (*P* ≤ 0.05) 205 d BW compared to their counterparts with the lowest (*P *= 0.04) 205 d BW calves weaned by Late May-born cows. Pregnancy rate as a 2-yr-old was influenced (*P* = 0.03) by previous treatment groups as heifers. Convert cows had the greatest (*P* < 0.01) pregnancy rate compared to May-born cows; however, pregnancy rate was not different (*P* = 0.34) between Early and Late May-born cows.

**Table 3.** Impact of converting March-born heifers in a May-calving herd on cow-calf performance as a 2-yr-old

	Treatment^1^				
Item	Convert	Early	Late	SEM	*P*-value
n =	79	98	49	--	*--*
Cow BW, kg					
Pre-calving	418^a^	396^b^	392^b^	5	< 0.01
Pre-breeding	408^a^	385^b^	391^b^	6	< 0.01
Weaning	408^a^	392^b^	394^b^	7	0.10
Cow BCS					
Pre-calving	5.65^a^	5.46^b^	5.44^b^	0.05	< 0.01
Pre-breeding	5.33	5.25	5.23	0.06	0.29
Weaning	5.16	5.09	5.04	0.07	0.41
Calving date, Julian day	131	134	134	1.8	0.21
Calf BW, kg					
Birth	28	29	29	1	0.65
Pre-breeding	81^a^	75^b^	73^b^	2	< 0.01
Weaning	160^a^	152^b^	147^b^	3	< 0.01
205 d ^2^	195^a^	189^b^	180^c^	3	< 0.01
Pregnancy rate, %	85^a^	64^b^	73^b^	--	0.03

^a,b,c^Means with different superscripts differ (*P* < 0.05).

^1^Treatments = March-born heifers converted over into a May-calving herd (Convert), May-born heifers born in the first 21-d of the calving season (Early), and May-born heifers born after the first 21-d of the calving season (Late).

^2^Weaning weight adjusted to 205 d of age using Beef Improvement Federation adjustments without age of dam or calf sex.

### Impact on Performance as a 3-yr-Old

Calving date as a 3-yr-old cow was not influenced (*P* = 0.43; Table 4) by previous heifer treatment groups. In contrast to cow BW as a 2-yr-old, cow BW was not influenced (*P* ≥ 0.51) at pre-calving, pre-breeding, and weaning by previous heifer treatment groups. Cow pre-calving BCS was not influenced (*P* = 0.63) by previous heifer treatment groups. However, pre-breeding BCS tended (*P* = 0.09) to be greater for Early May-born cows than Convert or Late May-born cows. At weaning, Early and Late May-born cows had greater (*P* = 0.01) BCS than Convert cows. Calf BW at birth was not influenced (*P* = 0.42) by previous heifer treatment groups. In contrast, calf pre-breeding BW tended (*P *= 0.09) to be greater for calves from Convert cows with no difference (*P* = 0.84) between calves from Early and Late May-born cows. However, calf BW at weaning and adjusted 205 d BW was not influenced (*P* ≥ 0.32) by previous heifer treatment groups. Pregnancy rate of 3-yr-old cows was not influenced (*P* = 0.91) by previous heifer treatment groups.

**Table 4.** Impact of converting March-born heifers in a May-calving herd on cow-calf performance as a 3-yr-old

	Treatment^1^				
Item	Convert	Early	Late	SEM	*P*-value
n =	68	63	35	--	*--*
Cow BW, kg					
Pre-calving	469	465	467	8	0.91
Pre-breeding	462	468	457	10	0.61
Weaning	439	450	443	11	0.51
Cow BCS					
Pre-calving	5.64	5.69	5.73	0.07	0.63
Pre-breeding	5.60^b^	5.76^a^	5.66^b^	0.08	0.09
Weaning	5.19^a^	5.45^b^	5.41^b^	0.09	0.01
Calving date, Julian day	144	143	149	4	0.43
Calf BW, kg					
Birth	31	31	32	1	0.42
Pre-breeding	88^a^	81^b^	81^b^	4	0.09
Weaning	178	170	171	5	0.32
205 d ^2^	222	216	215	5	0.38
Pregnancy rate, %	96	100	100	--	0.91

^a,b,c^Means with different superscripts differ (*P* < 0.05).

^1^Treatments = March-born heifers converted over into a May-calving herd (Convert), May-born heifers born in the first 21-d of the calving season (Early), and May-born heifers born after the first 21-d of the calving season (Late).

^2^Weaning weight adjusted to 205 d of age using Beef Improvement Federation adjustments without age of dam or calf sex.

### Impact on Performance as a 4-yr-Old

Calving date was not influenced (*P* = 0.63; Table 5) by previous heifer treatment groups. In addition, cow BW at pre-calving, pre-breeding, and weaning were not different (*P* ≥ 0.20) among treatment groups. Pre-calving and pre-breeding BCS were not different (*P* ≥ 0.84) among treatment groups. In contrast, cow BCS at weaning was influenced (*P* = 0.01) by heifer treatment groups. Convert cows were the thinnest (*P* = 0.01) with no difference between (*P* = 0.62) Early and Late May-born cows. Calf BW at birth was influenced (*P* = 0.04) by previous heifer treatment groups. Calves born from Convert cows tended (*P* = 0.07) to have greater BW at birth than calves from Early May-born cows and had increased (*P* = 0.02) BW compared to Late May-born cows; however, there were no differences (*P* = 0.31) in calf BW at birth between Early and Late-May born cows. In contrast, calf BW at pre-breeding and weaning were not influenced (*P* > 0.15) by previous heifer treatment groups. However, adjusted 205 d BW was influenced (*P* = 0.04) by previous heifer treatment groups. Adjusted 205 d BW was not different (*P* = 0.31) between calves from Convert cows and Late May-born cows, nor was there a difference (*P* = 0.37) between calves from Early and Late May-born cows; however, calves from Convert cows were larger (*P* = 0.01) than calves from Early May-born cows. Pregnancy rate as a 4-yr-old was not influenced (*P* = 0.46) by previous heifer treatment groups.

**Table 5.** Impact of converting March-born heifers in a May-calving herd on cow-calf performance as a 4-yr-old

	Treatment^1^				
Item	Convert	Early	Late	SEM	*P*-value
n =	65	62	35	--	*--*
Cow BW, kg					
Pre-calving	470	473	461	11	0.66
Pre-breeding	488	487	494	12	0.89
Weaning	469	481	488	12	0.20
Cow BCS					
Pre-calving	5.40	5.35	5.42	0.11	0.86
Pre-breeding	5.60	5.66	5.65	0.09	0.84
Weaning	4.93^b^	5.24^a^	5.32^a^	0.13	0.01
Calving date, Julian day	145	143	141	3	0.63
Calf BW, kg					
Birth	35^a^	33^ab^	32^b^	2	0.04
Pre-breeding	84	81	81	6	0.60
Weaning	219	209	216	9	0.15
205 d ^2^	227^a^	216^b^	221^ab^	8	0.04
Pregnancy rate, %	88	95	100	--	0.46

^a,b,c^Means with different superscripts differ (*P* < 0.05).

^1^Treatments = March-born heifers converted over into a May-calving herd (Convert), May-born heifers born in the first 21-d of the calving season (Early), and May-born heifers born after the first 21-d of the calving season (Late).

^2^Weaning weight adjusted to 205 d of age using Beef Improvement Federation adjustments without age of dam or calf sex.

### Impact on Performance as a 5-yr-Old

Calving date as a 5-yr-old cow was not influenced (*P* = 0.31; Table 6) by previous heifer treatment groups. Cow BW at pre-calving, pre-breeding, and weaning were not influenced (*P* ≥ 0.31) by previous heifer treatment groups. Similarly, cow BCS at all time points was not influenced (*P* ≥ 0.38) by previous heifer treatment groups. In addition, calf BW at all weigh points was not different (*P* ≥ 0.68) among treatment groups. Pregnancy rate for 5-yr-old cows were not influenced (*P* = 0.22) by previous heifer treatment groups.

**Table 6.** Impact of converting March-born heifers in a May-calving herd on cow-calf performance as a 5-yr-old

	Treatment^1^				
Item	Convert	Early	Late	SEM	*P*-value
n =	57	59	35	--	*--*
Cow BW, kg					
Pre-calving	500	501	521	12	0.31
Pre-breeding	503	504	520	13	0.50
Weaning	470	480	490	13	0.53
Cow BCS					
Pre-calving	5.26	5.33	5.39	0.13	0.62
Pre-breeding	5.51	5.47	5.64	0.09	0.38
Weaning	4.96	4.98	5.16	0.14	0.48
Calving date, Julian day	142	139	139	2	0.31
Calf BW, kg					
Birth	35	35	36	1	0.85
Pre-breeding	87	89	90	4	0.75
Weaning	214	214	215	6	0.99
205 d ^2^	211	208	208	5	0.68
Pregnancy rate, %	92	97	83	--	0.22

^a,b,c^Means with different superscripts differ (*P* < 0.05).

^1^Treatments = March-born heifers converted over into a May-calving herd (Convert), May-born heifers born in the first 21-d of the calving season (Early), and May-born heifers born after the first 21-d of the calving season (Late).

^2^Weaning weight adjusted to 205 d of age using Beef Improvement Federation adjustments without age of dam or calf sex.

### Impacts on Long-Term Productivity

**Figure 1.**
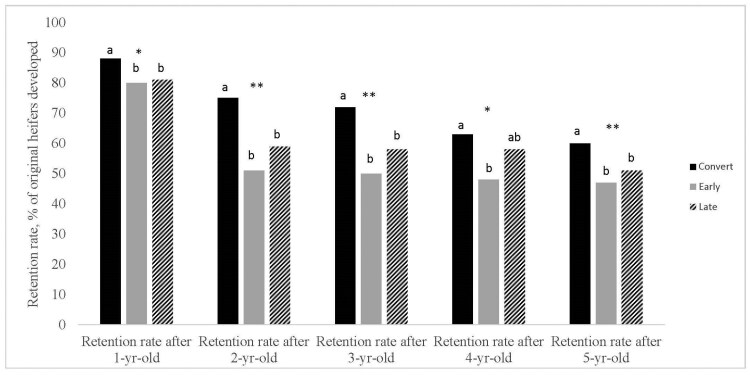
Retention rate of March-born heifers converted into a May-calving herd (Convert) compared to May-born heifers born in the first 21-d of the calving season (Early) or May-born heifers born after the first 21-d of the calving season (Late). Values shown in retention rate after 1-yr-old are heifer pregnancy rates. Values after retention rate after 1-yr-old represent the portion of original heifers remaining in the herd after each respective breeding season. ^a,b^Means with different superscripts differ (*P* ≤ 0.05). * and ** represent a tendency (*P* > 0.05 and *P* ≤ 0.10) or significant (*P* ≤ 0.05); respectively.

Heifer retention rates (heifer pregnancy rates; also shown in Table 1) tended (*P* = 0.08; Figure 1) to be greater for Convert heifers than their counterparts with no difference between Early and Late May-born heifers. Retention rates at 2 and 3 yr of age were different (*P* ≤ 0.05) among treatment groups with Convert cows having greater retention (*P* = 0.01) than their counterparts and no differences (*P* ≥ 0.66) between Early and Late May-born cows. Retention rate at 4 yr of age showed a tendency (*P* = 0.07) to be different among treatment groups. Retention rate was not different (*P* = 0.53) between Convert and Late May-born cows; however, Convert cows did have an increased (*P* = 0.05) retention rate compared to Early May-born cows. At 5 yr of age, retention rate was increased (*P* = 0.05) in Convert cows compared to Early and Late May-born cows with no difference (*P* = 0.84) between the two May-born heifer groups. Total kilograms of calf weaned was greater (*P* = 0.04; Table 7) in Convert cows than their counterparts with no differences between Early and Late May-born cows.

**Table 7.** Impact of converting March-born heifers in a May-calving herd on total kilograms of calf weaned

	Treatment^1^				
Item	Convert	Early	Late	SEM	*P*-value
Total kilograms of calf weaned, kg/cow	415^a^	307^b^	317^b^	43	0.04

^a,b,c^Means with different superscripts differ (*P* < 0.05).

^1^Treatments = March-born heifers converted over into a May-calving herd (Convert), May-born heifers born in the first 21-d of the calving season (Early), and May-born heifers born after the first 21-d of the calving season (Late).

## DISCUSSION

Replacement heifers play an integral part in sustaining herd size, which is necessary to replace culled cows, maintain herd size, and improve the genetics of the herd. However, heifer development is one of the largest expenses for beef cattle operations due to inherent opportunity and development costs for retaining heifers (Clark et al., 2005), which it can take up to 10 yr to pay off those development and opportunity costs in some situations (McFarlane et al., 2018). One limitation of heifer development research is limited considerations for long-term productivity and overall longevity in the cowherd. For instance, an increased heifer pregnancy rate does not necessarily equate to increased longevity in the cowherd (Mulliniks et al., 2013). Management decisions made during this time frame can have lifelong effects on the efficiency of the beef female (Endecott et al., 2013; Mulliniks et al., 2013). Therefore, producers need to be aware of important aspects of heifer development methods such as: 1) costs of developing heifers and 2) longevity of replacement heifers in the cow herd.

Managing considerations and development of beef heifers have evolved over the last 50 yr, which corresponds to the shifting from calving heifers at 3 yr of age to calving at 2 yr of age. This shift was due to breeding heifers to calve at 2 yr of age increased lifetime production per cow by increasing the number of calves produced (Pinney et al., 1972; Price and Wiltbank, 1978). Alternative to calving at 24 or 36 mo of age, Morrison et al. (1992) reported that breeding heifers to calve at 30 mo of age resulted in increased artificial insemination pregnancy rate and overall pregnancy rate compared to 24 mo of age. In the previous study, Morrison et al. (1992) summarized that in production systems with spring and fall calving herds, exposing heifers from the alternant calving season will increase BW at breeding and increase reproductive performance, especially in the more nutrient challenging season. However, holding heifers over for another 6 additional months could increase production costs above the benefit of increased pregnancy rates. In the current study, Convert heifers were 59 to 82 d older than their May-born counterparts, which Convert heifers were larger at the start of breeding and had increased pregnancy rates.

Heifer birth date has been shown to impact the percentage of heifers cycling prior to breeding and the timing of conception date with heifers born in the first 21 d of calving having a greater percentage of heifers cycling prior to breeding and an increased percentage of heifers calving in the first 21 d of the subsequent calving season (Funston et al. 2012). Research on heifer development in the Nebraska Sandhills has reported heifers from a March-calving herd had greater proportion pregnant and increase in the percentage of heifers calving within the first 21 d compared to May heifers (Springman et al., 2018). However, May-born heifers had no delay in puberty attainment prior to the breeding season and were heavier at the start of breeding compared to March-born heifers (Springman et al., 2018). In 2 different studies in the same herd as the current study, Crouch (2024) reported that 32% to 39% of March-born heifers and 67% to 85% of May-born heifers achieving puberty by the start of their respective breeding seasons (June vs August, respectively). Pregnancy rates have been shown to be greater for heifers bred on their third estrus compared to first estrus (Byerley et al., 1987). However, conclusions from Byerley et al. (1987) may be limited due to confounded by age and BW of heifers at the time of breeding, which are not a traditional practice. Heifers were 10.7 to 12.5 mo of age at breeding (1^st^ vs 3^rd^ estrous cycle), which is 2 to 4 mo younger than May-born heifers in the current study. In a review, Endecott et al. (2013) suggested that puberty and pregnancy data reported in the last 30 yr demonstrate a decreased impact of delayed puberty on pregnancy rates as once believed. In addition, Roberts et al. (2019) illustrated that pregnancy rates increase for heifers that achieve estrus prior to the start of the breeding season; however, number of estrous cycles prior to the start of breeding did not impact pregnancy rates.

Heifers that calve in the first 21 d of a calving season have been shown to have increased longevity in the cowherd (Cushman et al., 2013). In the current study, converting March-born heifers into a May-calving herd resulted in increased longevity compared to May-born counterparts. Heifers born in the first 21-d of the May-calving season has similar long-term performance as late-born May heifers, which contrasts with Cushman et al. (2013). The differences may be due to differences in forage quality dynamics in the breeding season. Understanding forage growth and quality patterns along with nutrient requirement of beef cattle can influence producer management decisions. In the Nebraska Sandhills, summer calving seasons have become more prevalent due to reduced winter supplementation costs, calving labor cost, and calf death loss due to March/April snowstorms. In addition, May-calving cows in the Nebraska Sandhills have an advantage to March-calving cows due to calving in a greater plane of nutrition postpartum. However, forage quality starting in July and throughout the breeding season is often nutrient deficient leading to negative energy balance prior to breeding and decreased reproductive performance, especially in yearlings and young cows. With that in mind, rebreeding percentage of May-calving young cows (2- and 3-yr-olds) can be as low as 60% (Beard, 2020), which align with the pregnancy rates as 2-yr-old for the Early and Late May-born cows in the current study.

Although pregnancy loss or embryonic loss (early or late) was not measured, embryonic loss could be one factor influencing the differences in heifer pregnancy rates and rebreeding pregnancy rates as a 2-yr-old. Short-term changes in energy intake post-insemination have been shown to impact embryo survival. By altering grazing forage allowance to achieve maintenance energy requirement or restricted energy requirement pre- and post-breeding, Dunne et al. (1999) reported that heifers going from meeting energy requirements to a restricted energy intake post-breeding resulted in decreased embryo survival compared to heifers that either met maintenance energy requirement pre- and post-breeding or heifers were on a restricted energy requirements pre- and post-breeding. Maternal nutrient restriction at the start of breeding increases the risk of embryo loss in lactating beef cows (Serrano-Pérez et al., 2020). In the current study, forage quality (Table 1) has a sharp decline starting in June and approaches 6% to 7% crude protein at the start of breeding in August, which would be considered low-quality forages. This decline in forage quality will impact microbial activity and can subsequently decrease forage intake (Mathis, 2003). Therefore, the decline in energy intake going into and throughout the breeding season may potentially cause an increase in early embryonic loss, especially in lighter BW, younger heifers that have decrease forage intake capacity. The potential benefit of Convert heifers being larger and having a larger intake capacity may result in the increased overall pregnancy rate as yearling heifers.

Historically, cow lifetime productivity per cow as measured by increasing the number of calves produced was increased by shifting first time calving from 3 yr of age to 2 yr of age (Pinney et al., 1972; Price and Wiltbank, 1978). In the current study, Convert cows had increased kilograms of calf weaned up to weaning a calf at 5 yr of age compared to their May-born counterparts. The increased kilograms of calf weaned was driven by increased pregnancy rates as heifers and 2-yr-old, which increased long-term retention rate, and 2-yr-old Convert cows weaned larger calves than their counterparts. The increased calf BW at weaning may be due to an increase in milk production. Heifers calving at 2 yr of age produced approximately 25% less milk than heifers calving for the first time at 3 yr of age (Galindo-Gonzalez et al., 2006). Although grazing range intake was not measured, the increased cow BW early in life (up to pre-breeding as a 2-yr-old) in Converts may have allowed for increased forage intake, which is approximately 0.34 kg/d increase in dry matter intake (DMI; NASEM, 2016) prior to calving as a 2-yr-old. The increased pre-calving BCS and pregnancy rates in Convert cows may have been the result of the increased DMI. The most challenging reproductive time period for a cow is during the 2- and potentially 3-yr-old time period, which generally have the lowest pregnancy rates (Meek et al., 1999). A driver of the decrease in performance is the inability to consume enough energy to meet the demands of lactation, growth, and reproduction (Mulliniks and Beard, 2019). Therefore, the increase in cow herd retention and total kilograms of calf weaned in Convert cows may have been driven by increase in BW, BCS, and potentially DMI as a 2-yr-old.

## APPLICATIONS

Converting spring-born heifers (March) into a summer-calving herd (May) resulted in increased longevity and productivity compared to summer-born heifers. Selecting heifers born in the first 21 d of the May calving season did not result in increased reproductive performance or kilograms of calf weaned. In resource-limited breeding seasons, cow-calf producers may want consider purchasing heifers from earlier calving herds from similar environments and management to increase long-term profitability.
